# Optimization of Anthocyanin Extraction from Purple Sweet Potato Peel (*Ipomea batata*) Using Sonotrode Ultrasound-Assisted Extraction

**DOI:** 10.3390/foods14152686

**Published:** 2025-07-30

**Authors:** Raquel Lucas-González, Mirian Pateiro, Rubén Domínguez-Valencia, Celia Carrillo, José M. Lorenzo

**Affiliations:** 1 Institute for Agri-Food and Agri-Environmental Research and Innovation, Miguel Hernández University (CIAGRO-UMH), Ctra. Beniel km 3.2, 03312 Orihuela, Alicante, Spain; raquel.lucasg@umh.es; 2 Centro Tecnológico de la Carne de Galicia, Avd. Galicia N° 4, Parque Tecnológico de Galicia, 32900 San Cibrao das Viñas, Ourense, Spain; mirianpateiro@ceteca.net (M.P.); rubendominguez@ceteca.net (R.D.-V.); 3 Área de Nutrición y Bromatología, Facultad de Ciencias, Universidad de Burgos, Plaza Misael Bañuelos s/n, 09001 Burgos, Castile and León, Spain; ccarrillo@ubu.es; 4 Universidade de Vigo, Área de Tecnoloxía dos Alimentos, Facultade de Ciencias, 32004 Ourense, Galicia, Spain

**Keywords:** co-products, acylated anthocyanins, RSM-BBD, pigments, antioxidant activity, valorisation

## Abstract

Sweet potato is a valuable root due to its nutritional benefits, health-promoting properties, and technological applications. The peel, often discarded during food processing, can be employed in the food industry, supporting a circular economy. Purple sweet potato peel (PSPP) is rich in anthocyanins, which can be used as natural colourants and antioxidants. Optimising their extraction can enhance yield and reduce costs. The current work aimed to optimize anthocyanin and antioxidant recovery from PSPP using a Box-Behnken design and sonotrode ultrasound-assisted extraction (sonotrode-UAE). Three independent variables were analysed: extraction time (2–6 min), ethanol concentration (35–85%), and liquid-to-solid ratio (10–30 mL/g). The dependent variables included total monomeric anthocyanin content (TMAC), individual anthocyanins, and antioxidant activity. TMAC in 15 extracts ranged from 0.16 to 2.66 mg/g PSPP. Peonidin-3-caffeoyl-p-hydroxybenzoyl sophoroside-5-glucoside was the predominant anthocyanin. Among four antioxidant assays, Ferric-reducing antioxidant power (FRAP) showed the highest value. Ethanol concentration significantly influenced anthocyanin and antioxidant recovery (*p* < 0.05). The model demonstrated adequacy based on the coefficient of determination and variation. Optimal extraction conditions were 6 min with 60% ethanol at a 30 mL/g ratio. Predicted values were validated experimentally (coefficient of variation <10%). In conclusion, PSPP is a promising matrix for obtaining anthocyanin-rich extracts with antioxidant activity, offering potential applications in the food industry.

## 1. Introduction

The food industry continually addresses challenges to offer safe, healthy, tasty, and attractive foods to consumers. In recent decades, consumers have become more aware of the connection between health and diet while also raising awareness about the importance of protecting the environment. As a result, they now demand clean labels and sustainable manufacturing processes [1,2]. Consequently, the food industry aims to replace synthetic additives with natural alternatives, decreasing the number of additives used and innovating in manufacturing processes.

In this context, coproducts from the agri-food industry represent a valuable source of natural additives. Many of their nutrients and bioactive compounds exhibit properties such as colouring, antioxidant, antimicrobial, or emulsifying, which are useful for food manufacturing [3,4,5,6,7]. To recover many of these compounds, extraction processes are required. In line with the trends to minimize the environmental carbon footprint of manufacturing, emerging non-thermal technologies have been investigated for the extraction of bioactive compounds from vegetable matrices. Among these, ultrasonic-assisted extraction using sonotrode has gained considerable attention due to its efficiency and sustainability [6].

Sonotrode ultrasound-assisted extraction (sonotrode-UAE) significantly reduces extraction time while enabling the simultaneous recovery of both free and conjugated compounds, such as polyphenols. Furthermore, this method delivers high extraction efficiency without relying on petroleum-derived solvents such as methanol, acetone, or ethyl acetate, which are commonly used in phytochemical extraction processes [8]. Ultrasonic cavitation involves the formation, growth, and violent collapse of microbubbles within a liquid medium. This phenomenon generates intense mechanical forces, which disrupt cell walls and significantly enhance the release of bioactive compounds [9]. Additionally, sonotrode-UAE is a scalable technology that enables significantly higher recovery of polyphenols compared to conventional extraction methods [10,11]. For instance, Martín-García et al. [12] reported a 57.9% increase in total polyphenol content recovered from rice using sonotrode-UAE compared to traditional extraction.

The efficiency of the extraction process reflects not only effective resource utilization but also waste reduction. In this context, Response Surface Methodology (RSM) serves as a valuable tool for process optimization, helping to reduce time, workload, resource and energy consumption, as well as overall costs [13]. RSM is a widely used statistical tool for modelling and optimising processes influenced by multiple variables. It enables the identification of interactions between factors and their effects on responses, thereby reducing the number of experimental trials required. Among the various RSM designs, the Box–Behnken Design (BBD) is particularly effective for evaluating cause-and-effect relationships in experimental studies [14]. The integration of RSM with emerging extraction technologies represents a growing trend in the scientific community, aiming to optimize extraction efficiency while minimising environmental impact [13].

Sweet potato (*Ipomoea batatas* (L.) Lam.) is the third most harvested tuber crop globally, after potato and cassava, with 95 million tons produced in 2023. China remains the largest producer, contributing approximately 51 million tons. Additionally, several African countries, including Malawi, the United Republic of Tanzania, and Nigeria, collectively produced around 16.6 million tons in the same year [15]. The flesh and peel of the tuber can be white, orange, or purple. In recent years, sweet potato has gained popularity, particularly in the development of vegetable-based snack products. As the sweet potato industry continues to expand, a significant amount of peel waste is generated. The peel accounts for approximately 10–25% of the total tuber weight [16], leading to substantial waste on a global scale. Valorising these co-products is essential for promoting a circular economy and ensuring more sustainable agri-food manufacturing practices.

The colour of the tuber is closely associated with its phytochemical composition. For example, orange-fleshed and -peeled varieties are rich in carotenoids, while purple sweet potatoes are particularly high in anthocyanins. Over the past few decades, purple sweet potato has attracted growing interest due to its high nutritional value and its content of bioactive macro- and micronutrients, such as starch and anthocyanins, which have promising pharmaceutical and technological applications [17].

From a technological point of view, anthocyanins from purple sweet potato have been mainly used to prepare colorimetric indicator labels or colorimetric pH indicator films due to the ability of anthocyanins to change colour with pH variations, or as a natural colourant to foods like ice cream [18,19,20,21,22]. Moreover, purple sweet potatoes are rich in acylated anthocyanins, which exhibit higher antioxidant activity than their corresponding anthocyanidins and glycosides [23]. This makes purple sweet potatoes and their co-products particularly attractive as natural antioxidants and colourant additives for use in the meat, dairy, and bakery industries.

Anthocyanins are typically extracted from the purple sweet potato flesh, either fresh or dehydrated. Extraction is commonly performed with acidified hydroalcoholic solutions (40–96% ethanol), using solid-to-liquid ratios ranging from 1:7 to 1:20. The extraction process involves stirring, ultrasonication in an ultrasound bath, or maceration, generally at temperatures between 35 °C and 80 °C and durations ranging from 0.5 to 24 h. After extraction, the mixture is usually filtered or centrifuged, and the extract is concentrated using a rotary evaporator, which is then usually freeze-dried [21,22,24,25,26,27]. Purple sweet potato peel has also been used for anthocyanin extraction, though less frequently [16,28].

These extraction processes consume time and require high temperatures, which increases the carbon footprint and may reduce the process’s profitability. Therefore, sonotrode-UAE could help save time and energy [29]. According to the literature, several studies have applied sonotrode-UAE for the extraction of anthocyanins from fresh purple sweet potatoes. However, many of these approaches still rely on extended extraction times (e.g., 52 min) and high temperatures (around 68 °C) [30], which may counteract the benefits of sonotrode-UAE. Other authors used non-food-grade solvents, such as ammonium sulphate and McIlvaine buffer [18,31]. Moreover, most optimisation studies have primarily focused on maximising anthocyanin content, with limited attention to their antioxidant activity [22]. Therefore, there is also a lack of detailed information regarding how different extraction parameters affect the individual anthocyanin profile, which is crucial for assessing the quality and functionality of the extracts.

Therefore, in line with current trends toward eco-friendly products with a low carbon footprint, the present work aimed to optimize sonotrode-UAE to obtain an anthocyanin-based colouring-antioxidant extract from purple sweet potato peel in a green, efficient, and scalable manner to be used by the food industry. A Box-Behnken design was employed as RSM, using total anthocyanin content, anthocyanin profile, and antioxidant activity as dependent variables. Additionally, sonotrode-UAE efficiency was compared to magnetic agitation under optimised conditions.

## 2. Materials and Methods

### 2.1. Plant Material

**Figure 1:**
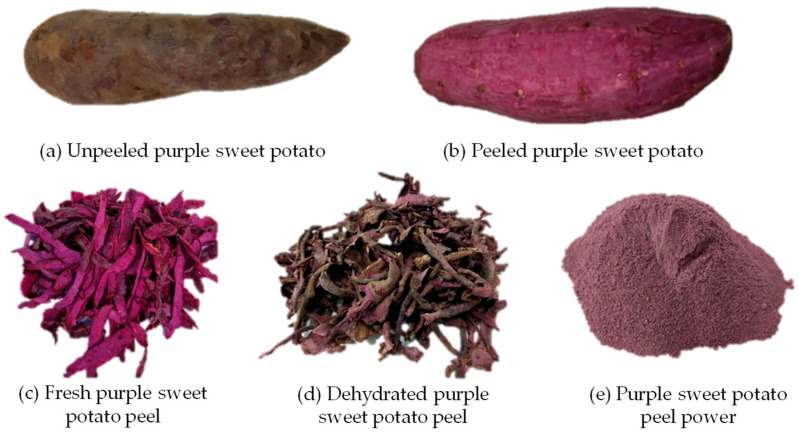
Image of purple sweet potato. (**a**) Unpeeled, (**b**) peeled, (**c**) fresh, (**d**) dehydrated, (**e**) milled and sieved (<1 mm).

Purple sweet potato (*Ipomoea batatas* (L.) Lam.) was purchased from a local commercial market (Valencia, Spain). Briefly, 750 kg of tubers were brushed for 8 min in an automatic potato dry-cleaning machine, followed by manual peeling. The peel accounted for 75 kg, representing a 10% recovery yield, and was subsequently dehydrated at 45 °C for 12 h. Then, 15 kg of dehydrated peel (20% recovery) was milled, sieved (particle size < 1 mm), and packed in individual plastic bags under vacuum (Figure 1). The samples were kept in refrigeration at 4 °C until their use.

### 2.2. Experimental Design

A Box-Behnken design (BBD) was employed to evaluate the effects of three independent variables, extraction time (minutes), liquid-to-solid ratio (mL/g), and ethanol concentration (%) on the dependent variables, including antioxidant activity (2,2-diphenyl-1-picrylhydrazyl (DPPH) radical assay, ferric-reducing antioxidant power (FRAP) assay, 2,2-azinobis-(3-ethyl-benzothiazoline-6-sulphonate (ABTS) radical cation decolourization assay, oxygen radical absorbance capacity (ORAC) assay), total monomeric anthocyanin content (TMAC), Total anthocyanin content (TAC) and anthocyanin profile of PSPP (Table 1).

BBD was selected due to its efficiency in reducing the number of experimental runs, especially in the case of three-variable optimization, while maintaining high prediction accuracy [13]. Response surface methodology (RSM) allowed the optimization of the anthocyanin extraction process through mathematical and statistical models [11,22]. The fit of the experimental data to polynomial equations allows establishing data behaviour and statistical predictions. Therefore, RSM-BBD allows for optimal design by requiring fewer experiments for process optimization [14].

The experimental design consisted of 15 total runs, including three replicates at the central point to estimate pure error and ensure the reliability of the model. Extraction runs were randomised to enhance accuracy and minimize systematic bias. Independent variables were tested at three levels: low (−1), central (0), and high (+1). The specific ranges can be observed in Table 2. RSM was applied to determine the optimal conditions of the significant factors influencing sonotrode-UAE and to develop a predictive model describing changes in the response as a function of extraction time, liquid-to-solid ratio, and ethanol concentration in water.

The experimental data were fitted to a second-order polynomial model, expressed as:

(1) $$Y=\beta o+\sum_{i=1}^{k}\beta_{i}X_{i}+\sum_{i=j}^{k}\beta_{ii}X_{i}^{2}+\sum \sum_{i<j}^{k}\beta_{ij}X_{i}X_{j}$$

where Y represents the dependent (response) variable, Xᵢ and Xⱼ are the independent variables (coded factor levels), β_0_ is the intercept, and βᵢ, βᵢⱼ, and βᵢᵢ are the regression coefficients for linear, interaction, and quadratic terms, respectively.

The experimental results were analysed using Statistics V8.0 (Statsoft Inc., Tulsa, OK, USA), which was employed to calculate regression coefficients and optimize the conditions for all responses. Model adequacy was evaluated based on the coefficient of determination (R^2^), analysis of variance (ANOVA), and coefficient of variation. The statistical significance of model terms was assessed using *p*-values (*p* < 0.05). To visualize the influence of the independent variables on the response. Response surface plots were generated.

### 2.3. Extraction of Anthocyanins

#### 2.3.1. Sonotrode-UAE

**Figure 2:**
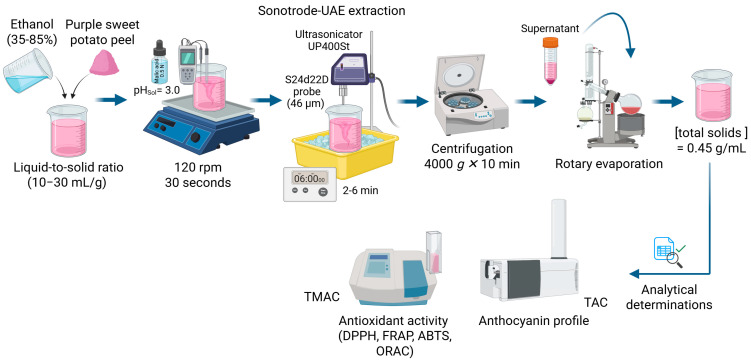
Schematic representation of anthocyanin extraction process using sonotrode-UAE (created with BioRender).

Anthocyanins were extracted from purple sweet potato peel (PSPP) using an ultrasonicator UP400St (Hielscher, Germany) operating at 400 W and 24 kHz. A S24d22D probe (46 µm) was used for sonotrode-UAE (Figure 2). To prevent excessive heat buildup and potential anthocyanin degradation, an ice bath was used to maintain the extraction temperature within the predetermined range (50 °C).

The independent variables evaluated were ethanol concentration (%), extraction time (minutes), and liquid-to-solid ratio (mL/g), as detailed in Table 1. The following extraction parameters were kept constant: ultrasonic power (180 W), amplitude (100%), extraction volume (180 mL), upper temperature limit (50 °C), delta temperature (10 °C), and solvent pH (3.0), adjusted using 1.0 N malic acid. Before sonication, samples were pre-homogenised in the solvent using magnetic stirring at 120 rpm for 30 s to ensure uniform extraction conditions. After the sonication treatment, samples were immediately centrifuged at 4000× *g* for 10 min at room temperature. The supernatant was then concentrated to dryness using a rotary evaporator (Büchi Rotavapor R-200, BUCHI Ibérica S.L.U, Barcelona, Spain) at 50 °C. The dried extracts were then resuspended in distilled water at a final concentration of 0.45 g/mL. Antioxidant activity (DPPH, FRAP, ABTS, ORAC), total monomeric anthocyanin content (TMAC), total anthocyanin content (TAC), and anthocyanin profile were determined in the obtained extracts. All extracts were stored at −80 °C until further analysis.

#### 2.3.2. Agitation

Magnetic agitation (150 rpm) was used to extract anthocyanins from PSPP, with the optimal conditions obtained using sonotrode-UAE (60% ethanol, 6 min, 30 mL/g).

### 2.4. Anthocyanin Content and Profile

#### 2.4.1. Total Monomeric Anthocyanin Content (TMAC)

The total monomeric anthocyanin content in PSPP extracts was determined using the pH-differential method described by Giusti and Wrolstad [32]. This method relies on the reversible structural changes of anthocyanins at different pH levels, which produce distinct absorbance spectra. Briefly, the extracts were diluted 1:5 with potassium chloride buffer (0.025 M; pH 1.0) and sodium acetate buffer (0.4 M; pH 4.5). After that, both dilutions were left to rest for 15 min. Absorbance was measured at 530 and 700 nm. Equation (1) was used to calculate TMAC in the studied extracts:

TMAC (mg/100 g) = [((A × MW × DF)/(ε × 1))/C] × 100(2)

where A: absorbance, MW: molecular weight of cyanidin-3-glucoside (449.2), DF: dilution factor, ε: molar absorptivity of cyanidin-3-glucoside (26,900), C: concentration of sample (g/mL).

The results were expressed as mg cyanidin-3-glucoside equivalent/g of PSPP.

#### 2.4.2. Detection and Identification of Anthocyanin Profile

Anthocyanin extracts were purified and concentrated before identification, following the methodology described by Lucas-González et al. [33]. In brief, a C18 solid phase extraction cartridge (CHROMAFIX^®^) was used. Firstly, cartridges were conditioned with water, methanol, and HCl (0.01 M). After that, an aliquot of the PSPP extract (5 mL) was passed through a cartridge, followed by pure distilled water to remove sugars and other impurities. Anthocyanins were eluted into cartridges with 1 mL of formic acid/methanol (1:99; *v*/*v*). After that, a polymer reversed phase column (PLRP-S; 250 × 4.6 mm, 5 µm particle size, 100 Å pore size; Agilent Technologies Inc., Palo Alto, CA, USA) was used to separate compounds following the analytical method described by Zoubiri et al. [34] with some modifications, which are described in Lucas-González et al. [33]. In brief, a linear gradient with 0.8 mL/min of solvent A, 0.1% formic acid in water and solvent B, and 0.1% formic acid in acetonitrile was used. An Agilent 1260 Infinity high-performance liquid chromatography system, coupled to a 6545 accurate-mass quadrupole Time-of-Flight (QTOF) with a Jet Stream Ionization source, was used to obtain the mass spectra of the compounds. Data were acquired in positive electrospray ionization mode as follows: gas nitrogen flow rate 10 L/min (400 °C); drying gas nitrogen flow rate 8 L/min (350 °C); nebulizer pressure 50 psi; nozzle voltage 1 kV; capillary voltage 3.5 kV. Signals were recorded in the m/z 100–1200 range. Calibration curves with cyanidin-3-O-glucoside were used to quantify the individual anthocyanins. TAC, expressed as mg of cyanidin-3-O-glucoside equivalents per g of PSPP, resulted from the sum of individual anthocyanin concentrations.

### 2.5. Determination of In Vitro Antioxidant Activity

To obtain a comprehensive assessment of antioxidant activity, four different in vitro antioxidant assays were performed on the different PSPP extracts, following the procedures described by Franco et al. [35].

#### 2.5.1. DPPH Radical Scavenging Assay

The methodology carried out was based on the previously published assay by Brand-Williams et al. [36]. In brief, the 2,2-diphenyl-1-picrylhydrazyl (DPPH) radical was diluted in methanol (60 µM). Then it was mixed with the sample (100 µL) and incubated at 37 °C for 10 min. The absorbance was measured in a spectrophotometer (UV-1800, Shimadzu Corporation, Kyoto, Japan) at 515 nm. A Trolox standard curve was submitted to the same assay as the samples. The results were expressed as mg Trolox equivalents (TE)/g PSPP.

#### 2.5.2. ABTS Radical Cation Decolourization Assay

The ABTS method followed the fundamentals proposed by Re et al. [37]. In brief, 20 μL of the 2,2-azinobis-(3-ethyl-benzothiazoline-6-sulphonate (ABTS) solution was added to the PSPP extract, and the resultant mixture was mixed and left in the dark for 10 min. Afterwards, absorbance was measured in a spectrophotometer (UV-1800, Shimadzu Corporation, Kyoto, Japan) at 734 nm. The results were expressed as mg ascorbic acid equivalents (AAE)/g PSPP.

#### 2.5.3. Ferric-Reducing Antioxidant Power (FRAP) Assay

The methodology proposed by Benzie and Strain [38] was employed. In brief, the FRAP reagent was prepared by mixing acetate buffer, TPTZ, and FeCl_3_·6H_2_O in a 10:1:1 ratio. A diluted sample was added to the reagent and incubated at 37 °C for 20 min. Absorbance was measured at 593 nm using a spectrophotometer (UV-1800, Shimadzu Corporation, Kyoto, Japan). Antioxidant activity was determined using a FeSO_4_ standard curve, with results expressed as μmol Fe^2+^/g PSPP.

#### 2.5.4. Oxygen Radical Absorbance Capacity (ORAC) Assay

The ORAC assay was determined according to the Huang et al. [39] method, with some modifications. In brief, the reaction was conducted in 75 mM phosphate buffer (pH 7.4) with fluorescein (80 nM) and pre-incubated at 37 °C. AAPH (2,20-azobis (2-methylpropionamidine) dihydrochloride solution (184 mM, final concentration)) was added, and fluorescence was recorded every minute for 150 min in a Synergy™ H4 Hybrid Multi-Mode Microplate Reader (BioTek Instruments, Inc., Winooski, VT, USA). Antioxidant activity was calculated using Trolox as a standard and expressed as mg Trolox equivalents (TE)/g PSPP.

### 2.6. Statistical Analysis

The results are expressed as the mean ± standard deviation of three replicates. The software STATISTICA 8.0 (Statsoft Inc., Tulsa, OK, USA) was used to analyse the results derived from BBD-RSM. The software SPSS (version 26.0, SPSS Inc., Chicago, IL, USA) was used to carry out an ANOVA (one-way) assay using a confidence level of 95% to determine any significant differences (*p* < 0.05) between anthocyanin content after sonotrode-UAE and magnetic agitation.

## 3. Results and Discussion

A Box–Behnken design with response surface methodology (BBD-RSM) was used to evaluate the effects of extraction time (×1), liquid-to-solid ratio (×2), and ethanol concentration (×3) on anthocyanin recovery and antioxidant activity from PSPP via sonotrode-UAE. The optimization process contributes to maximising extraction efficiency while minimising energy and time cost. Furthermore, sonotrode-UAE can enhance the process by reducing extraction time and the use of petroleum-derived solvents. This makes it a promising technique for developing new natural colourants and antioxidants through environmentally friendly methods. Although several studies have optimised anthocyanin extraction from purple sweet potato [18,40], to the best of our knowledge, this is the first study to utilize the peel as a co-product to obtain an anthocyanin-rich extract with both colourant and antioxidant activity.

### 3.1. Effect of Sonotrode-UAE on Anthocyanin Content

#### 3.1.1. Effect of Sonotrode-UAE on TMAC

The total monomeric anthocyanin content (TMAC) in the 15 evaluated extracts ranged from 0.16 to 2.66 mg/g PSPP. The best extraction conditions resulted in a TMAC that was 15.6 times higher than the lowest value observed. The highest TMAC was achieved using a 60% ethanol solution with a liquid-to-solid ratio of 30 (mL/g) and an extraction time of 6 min (Run 4). In contrast, the lowest TMAC was observed in the extract obtained under the following conditions: 85% ethanol, a liquid-to-solid ratio of 20 (mL/g), and an extraction time of 2 min (Run 7). These results highlighted the significant impact of extraction conditions on the recovery of bioactive compounds. Run 7 showed the highest pH value (5.30), which may have contributed to the degradation of anthocyanins. However, pH alone does not explain all the variations in TMAC. A Pearson correlation analysis between extract pH and TMAC showed a negative trend, but it was not statistically significant (R = −0.513; *p* = 0.060).

TMAC obtained under optimal extraction conditions was higher compared to previous studies. For instance, Ke et al. [41] used whole dehydrated purple sweet potato and obtained 1.36 mg/g DW after 4 h of extraction with a 70% ethanol aqueous solution (1:10, *w*/*v*) at 40 °C. Similarly, Zhao et al. [16] applied a 24 h extraction method, obtaining 0.513 mg/g DW, by blending PSPP powder with an 80% ethanol aqueous solution (1:20, *w*/*v*), followed by ultrasonication at 400 W for 40 min at 4 °C. Zhu et al. [28] reported 0.393 mg/g from powder peel extracted with 70% EtOH in an ultrasound bath after two extraction cycles. Differences in variety, specific parts of the plant used, pre-treatment steps, and extraction conditions are likely responsible for the variations in TMAC observed in purple sweet potato [42,43].

**Figure 3:**
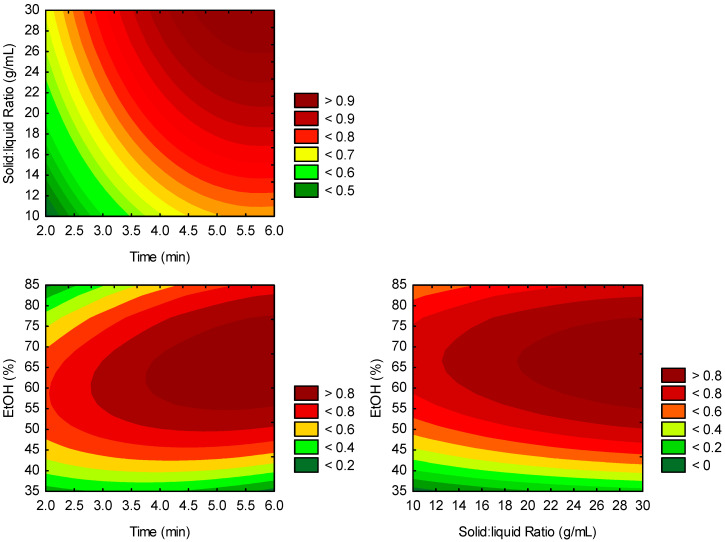
Contour plot as function of extraction time (min), liquid-to-solid ratio (mL/g), and ethanol concentration (%).

ANOVA results (Table 3) indicated that only ethanol concentration significantly affects TMAC recovery (*p* < 0.05). The linear term was positive, while the quadratic term was negative. Therefore, increasing ethanol from 35% to 72.5% enhanced recovery, but further increases led to a decline. This effect can be appreciated in the Contour plot (Figure 3). Li et al. [44] also reported that ethanol concentration was the only significant linear factor affecting anthocyanin yield from purple sweet potato flesh, among the parameters studied—extraction time, temperature, and liquid-to-solid ratio. However, the optimum concentration of the acidified ethanolic solution ranged between 76.7% and 82.25%.

The R^2^ value of 0.877 indicates that the model can predict 87.7% of the variability in TMAC (Table 3). However, other factors such as pH or temperature may also significantly influence anthocyanin recovery and should be considered to improve the model’s accuracy. For instance, Rodríguez-Mena et al. [18] reported that pH was the most influential parameter in optimising sonotrode-UAE anthocyanin recovery from purple sweet potato flesh, where pH, amplitude, and time were selected as independent variables. In contrast, Zhu et al. [30] found that temperature was the only significant factor affecting TMAC recovery after sonotrode-UAE. The final equations that predict the behaviour of the extraction system are shown in Table 4, where only significant values were included.

#### 3.1.2. Effect of Sonotrode-UAE on Anthocyanin Profile

Seventeen anthocyanins were identified in PSPP extracts (Table A1). The main anthocyanins present in PSPP were peonidin-3-caffeoyl-p-hydroxybenzoyl sophoroside-5-glucoside, which represented around 50% of the total anthocyanins quantified, followed by cyanidin-3-caffeoyl-p-hydroxybenzoyl-sophoroside-glucoside and peonidin-3-hydroxybenzoylsophoroside-5-glucoside. These results are consistent with previous studies, which report that the main anthocyanins in purple sweet potato are 3,5-diglucoside derivatives of cyanidin or peonidin, acylated with p-hydroxybenzoic acid, ferulic acid, or caffeic acid [42,43,45]

TAC ranged from 0.04 to 3.07 mg/g PSPP, showing a similar trend to TMAC. The highest (Run 4) and lowest (Run 7) TAC recoveries were observed in the same runs, with Run 4 yielding approximately 75 times more TAC than Run 7. These results highlight the significant impact of extraction conditions on anthocyanin recovery and the overestimation of the spectrophotometric method when low concentrations of anthocyanins are present in the extracts. Regarding the three main anthocyanins present in PSPP, the same trend in the highest and lowest recovery after BBD-RSM was reported, as can be expected (Table 1).

ANOVA results, like those observed for TMAC, revealed a significant quadratic effect of ethanol concentration on the recovery of the three main anthocyanins and total anthocyanin content (TAC) (Table 3). Increasing ethanol concentration from 35% to 72.5% enhanced anthocyanin recovery, but further increases led to a decline. This trend was also observed for most of the other quantified anthocyanins in PSPP, except for peonidin-3-caffeoyl-sulphide, which showed maximum recovery at 60% ethanol. In contrast, cyanidin-3-sophoroside-glucoside and peonidin-3-feruloyl-sophoroside-5-glucoside reached their highest recoveries at 85% ethanol. Similar results have been previously reported by Belwal et al. [11], who observed that ethanol concentrations of 35 and 65% showed higher cyanidin-3-O-galactoside extraction yield, while further increasing ethanol concentration resulted in a significant decrease in cyanidin-3-O-galactoside extraction yield from *Pyrus communis* ‘Starkrimson’ fruit peel [11].

Interestingly, in Runs 7 and 10, cyanidin-3-caffeoyl-p-hydroxybenzoyl-sophoroside-glucoside, the second most abundant anthocyanin in the other extracts, was not detected, highlighting the significant influence of extraction parameters (Table S1). A detailed comparison of glycosylated and acylated anthocyanins in PSPP revealed that Runs 5, 7, and 10 had low concentrations of diacylated anthocyanins (ranging from 8.65 to 34.03%) and higher levels of monoacylated anthocyanins (between 60.75 and 83.77%). In contrast, runs 4, 8, and 13, which showed the highest TAC, exhibited a higher proportion of diacylated anthocyanins (74.44–77.27%) and lower content of monoacylated anthocyanins (20.33–23.67%) (Table S1). This observation aligns with previous findings by Zhao et al. [46], who reported that the acylation decreases the water solubility of anthocyanins. Furthermore, it has been shown to effectively permeate plant tissue, exhibiting strong affinity and high solubility for anthocyanins [47].

The model presented strong accuracy by predicting TAC, peonidin-3-caffeoyl-p-hydroxybenzoyl sophoroside-5-glucoside, and cyanidin-3-caffeoyl-p-hydroxybenzoyl-sophoroside-glucoside, since the 93.3, 94.1, and 93.8%, respectively, can be predicted with the studied parameters. The prediction precision was satisfactory for peonidin-3-hydroxybenzoylsophoroside-5-glucoside, with an R^2^ value of 0.87. Additionally, the secondary-polynomial model equations established for TAC and the three main anthocyanins can be observed in Table 4.

### 3.2. Effect of Sonotrode-UAE on In Vitro Antioxidant Activity

Four in vitro antioxidant assays were conducted to evaluate the antioxidant activity of the 15 extracts obtained. The lowest antioxidant values were observed in the DPPH and ABTS methods, which measure the free radical scavenging capacity. DPPH values ranged from 5.97 to 18.67 mg TE/g, while ABTS values ranged from 7.30 to 17.12 mg AAE/g. In contrast, the FRAP assay yielded the highest values, ranging from 53.60 to 204.31 µmol Fe^2+^/g, indicating strong reducing power. The ORAC values ranged between 23.58 and 57.36 mg TE/g. In all four assays, Run 4 showed the highest antioxidant activity, while Run 7 had the lowest, following the same trend observed in anthocyanin content and profile (Table 5).

The results obtained in this study were higher than those reported by Im et al. [48] in different purple sweet potato varieties, where DPPH and ABTS values ranged around 12.0 mg TE/g and 6.0–8.0 mg TE/g, respectively. The antioxidant activity observed in PSPP extracts was generally lower compared to that reported in other anthocyanin-rich plants, such as elderberry (*Sambucus nigra* L.). However, FRAP values were notably higher in PSPP extracts than in elderberry, suggesting a stronger ferric reducing capacity. These differences in antioxidants could be attributed to the different anthocyanin profiles and the presence of different polyphenolic compounds in elderberry [49].

Regarding the ANOVA results for the four antioxidant activity assays, a significant negative quadratic effect of ethanol concentration was observed, like the trend found for TMAC, but with peak antioxidant activity occurring at lower ethanol concentrations. Specifically, antioxidant activity increased with ethanol concentrations between 35% and 60%, then declined at higher levels. These results suggest that, in addition to anthocyanins, other polyphenolic compounds contribute to the antioxidant activity and may have different solubility characteristics. In purple sweet potato, various hydroxycinnamic acids and their derivatives have been identified, including chlorogenic acid, 3-O-caffeoylquinic acid, 4,5-di-caffeoylquinic acid (di-CQA), and caffeic acid derivatives, among others [42,50]. Previous studies have shown that ethanol concentration negatively affects the extraction of hydroxycinnamic acids in other plant matrices, such as date palm seeds, as demonstrated using a Plackett–Burman design [8].

The CV (Table 3) in all reported antioxidant values was below 10%, indicating the solid prediction of the model [51].

### 3.3. Process Optimization and Validation of the Model

Since the main goal was to obtain an extract with high anthocyanin content and antioxidant activity, optimization was performed using TMAC, the three main anthocyanins, and four antioxidant activity assays as response variables. The central conditions selected for the BBD were 4 min of extraction time, a liquid-to-solid ratio of 20 mL/g, and 60% ethanol concentration. Based on the model predictions, the optimal extraction conditions were determined to be 6 min, 60% ethanol, and a liquid-to-solid ratio of 30 mL/g.

As shown in Table 5, all response variables exhibited a CV below 10%, with DPPH, ABTS, and TMAC reporting the lowest variability, with CV values below 5%. These low CV values indicate that the model demonstrates good reliability and predictive accuracy [51]. Similar CV ranges have been reported by other authors when optimising anthocyanin extraction using BBD-RSM [49].

Based on the strong anthocyanin recovery achieved (compared to previous studies) and the accuracy of the predictive model, we developed an encapsulated extract using spray-drying with maltodextrin, and its colouring and antioxidant properties were evaluated in beef burgers, as previously published [52]. Briefly, the resulting powder retained the same anthocyanin profile as the original extract and showed high total anthocyanin recovery (approximately 80–90% TAC). However, individual compounds such as cyanidin-3-sophoroside-glucoside and peonidin-3-sophoroside-5-glucoside exhibited lower recoveries, around 35% and 60%, respectively. The antioxidant activity of the encapsulated extract remained comparable to that of the non-encapsulated extract. Furthermore, the encapsulated extract effectively prevented lipid and protein oxidation and maintained colour stability in beef burgers during storage.

### 3.4. Comparison with Magnetic Agitation Method

The optimal extraction conditions (6 min, 60% ethanol, liquid-to-solid ratio of 30 mL/g) determined for sonotrode-UAE were also applied to an extraction using magnetic agitation for comparison. Overall, sonotrode-UAE yielded significantly higher anthocyanin contents than the magnetic agitation method (*p* < 0.05) (Table 6). Similarly, antioxidant activity was also significantly greater with sonotrode-UAE (*p* < 0.05). These results could be due to two main facts. First, anthocyanins are water-soluble polyphenols that are often stored within vacuoles of plant cells [53]. Therefore, in a milled matrix, where the cell walls have already been disrupted, they do not require extremely strong extraction forces for release. This contrasts with conjugated or bound polyphenols, which are more tightly associated with the wall cell and often require more intense physical disruption or chemical hydrolysis to be effectively extracted [8]. On the other hand, the differences in antioxidant activity may be attributed to the presence of other polyphenols, as previously mentioned, in addition to anthocyanins, which are likely extracted in greater quantities under sonotrode-UAE conditions.

In terms of anthocyanin composition, the most notable differences between the two extraction methods were observed for diacylated anthocyanins (*p* < 0.001) and monoacylated anthocyanins (*p* < 0.05), whereas no significant differences were found for glycosylated anthocyanins (*p* > 0.05). These findings suggest that the lower solubility of acylated anthocyanins may necessitate greater extraction forces to facilitate their diffusion from the cell matrix into the solvent. This requirement can be effectively addressed by the cavitation energy generated during ultrasonication, which promotes cell wall disruption and enhances the release and diffusion of less soluble acylated compounds. In contrast, glycosylated anthocyanins, which are typically more water-soluble and probably exist freely within vacuoles, can be extracted with less mechanical force.

## 4. Conclusions

The peel of sweet potato (PSPP) is rich in anthocyanins and exhibits significant antioxidant activity. The developed optimization model proved to be reliable in predicting both anthocyanin content and antioxidant capacity. The optimal extraction conditions for obtaining an antioxidant- and colourant-rich anthocyanin extract from purple sweet potato peel were 6 min of extraction, 60% ethanol concentration, and a liquid-to-solid ratio of 30 mL/g. Among the independent variables tested, ethanol concentration was the most significant factor, and anthocyanin recovery improved as ethanol concentration increased from 35% to 72.5%. A similar trend was observed across the four antioxidant assays; however, the peak antioxidant activity occurred at 60% ethanol. Ethanol concentrations beyond this range led to a decrease in anthocyanin yields and antioxidant activity, emphasising the importance of precise ethanol concentration control during extraction.

The study also revealed that extraction efficiency depends on the chemical structure of the compounds, with acylated anthocyanins requiring greater extraction force compared to glycosylated anthocyanins. Additionally, sonotrode-UAE demonstrated superior extraction efficiency over conventional methods such as magnetic stirring, achieving favourable results in a significantly shorter time.

Although differences between extraction methods were observed, further research is needed to optimize sonotrode parameters, such as amplitude, transmission energy, and power. Additionally, incorporating other factors like pH could be beneficial in achieving maximum yield.

In conclusion, the results demonstrate that a rich extract of anthocyanins with strong antioxidant activity can be efficiently obtained from purple sweet potato co-products in a short time using non-thermal emergent technologies and food-grade solvents. This approach offers promising potential for the food industry as a natural colourant and antioxidant, as previously demonstrated, derived from a sustainable source that avoids petroleum-based solvents. This promotes clean-label development, supports a circular economy, and increases the value of agricultural waste.

## Figures and Tables

**Table 1:** Experimental variables involved in the study.

Variable	Definition and Units	Nomenclature	Value/Range
Fixed	pH		3.0
	Power		180 W
	Probe		S24d22D
	Amplitude		46 µm; 100%
	Upper temperature limit		50 °C
Independent	Extraction Time (min)	t (x_1_)	2–6
	Liquid-to-solid ratio *v*/*w*	*v*/*w* (x_2_)	10–30 mL/g
	Ethanol concentration (*v*/*v* %)	EtOH (x_3_)	35–85%
Dependent	Total monomeric anthocyanin content (mg/g PSPP)	TMAC (y_1_)	
	Total anthocyanin content (mg/g PSPP)	TAC (y_2_)	
	Peonidin-3-caffeoyl-p-hydroxybenzoyl sophoroside-5-glucoside (mg/g)	Peo-3-ch (y_3_)	
	Cyanidin-3-caffeoyl-p-hydroxybenzoyl-sophoroside-glucoside (mg/g)	Cy-3-ch (y_4_)	
	Peonidin-3-hydroxybenzoylsophoroside-5-glucoside (mg/g)	Peo-3-h (y_5_)	
	ABTS (mg ascorbic acid equivalents/g PSPP)	ABTS (y_6_)	
	DPPH (mg Trolox equivalents/g PSPP)	DPPH (y_7_)	
	FRAP (µmol Fe^2+^/g PSPP)	FRAP (y_8_)	
	ORAC (mg Trolox equivalents/g PSPP)	ORAC (y_9_)	

**Table 2:** Box-Behnken design (expressed in terms of dimensional and dimensionless independent variables) and experimental results obtained for dependent variables.

	Independent Variables			Dependent Variables								
				TMAC, TAC, and Main Anthocyanins (mg/g PSPP)					Antioxidant Activity (mg/g PSPP)			
	t	*v*/*w*	EtOH	TMAC	TAC	Peo-3-ch	Cy-3-ch	Peo-3-h	ABTS	DPPH	FRAP	ORAC
No	x1	X_2_	X_3_	Y_1_	Y_2_	Y_3_	Y_4_	Y_5_	Y_6_	Y_7_	Y_8_	Y_9_
1	2 (−1)	10 (−1)	60 (0)	1.54 ± 0.00	1.65 ± 0.00	0.80 ± 0.01	0.18 ± 0.00	0.19 ± 0.00	11.40 ± 0.94	11.20 ± 2.01	132.31 ± 3.78	31.71 ± 0.96
2	6 (+1)	10 (−1)	60 (0)	1.67 ± 0.06	2.12 ± 0.02	1.04 ± 0.01	0.27 ± 0.00	0.22 ± 0.00	11.86 ± 0.09	13.75 ± 0.04	150.53 ± 4.45	37.41 ± 0.47
3	2 (−1)	30 (+1)	60 (0)	2.44 ± 0.08	2.68 ± 0.01	1.29 ± 0.01	0.36 ± 0.00	0.27 ± 0.00	15.74 ± 0.56	15.13 ± 1.80	190.35 ± 2.63	45.38 ± 3.80
4	6 (+1)	30 (+1)	60 (0)	2.40 ± 0.11	3.07 ± 0.07	1.47 ± 0.03	0.41 ± 0.01	0.31 ± 0.00	15.91 ± 0.00	18.16 ± 0.36	203.61 ± 4.50	52.68 ± 1.32
5	2 (−1)	20 (0)	35 (−1)	0.32 ± 0.00	0.27 ± 0.00	0.06 ± 0.00	0.00 ± 0.00	0.12 ± 0.00	9.86 ± 1.61	8.53 ± 0.43	58.01 ± 2.24	31.72 ± 0.00
6	6 (+1)	20 (0)	35 (−1)	0.63 ± 0.01	0.57 ± 0.01	0.23 ± 0.01	0.01 ± 0.00	0.15 ± 0.00	11.98 ± 0.81	13.60 ± 0.00	108.88 ± 12.49	33.73 ± 0.00
7	2 (−1)	20 (0)	85 (+1)	0.16 ± 0.01	0.04 ± 0.01	0.00 ± 0.00	0.00 ± 0.00	0.03 ± 0.00	8.96 ± 1.20	5.97 ± 0.39	53.60 ± 7.20	25.28 ± 0.63
8	6 (+1)	20 (0)	85 (+1)	2.20 ± 0.07	2.71 ± 0.02	1.28 ± 0.01	0.44 ± 0.00	0.25 ± 0.00	13.29 ± 0.50	13.40 ± 1.12	173.26 ± 7.45	35.51 ± 2.24
9	4 (0)	10 (−1)	35 (−1)	0.38 ± 0.01	0.41 ± 0.00	0.15 ± 0.00	0.01 ± 0.00	0.11 ± 0.00	7.30 ± 0.76	6.83 ± 0.57	61.67 ± 11.42	23.58 ± 2.79
10	4 (0)	30 (+1)	35 (−1)	0.45 ± 0.01	0.30 ± 0.02	0.06 ± 0.00	0.00 ± 0.00	0.13 ± 0.00	11.67 ± 0.79	12.57 ± 0.93	122.56 ± 8.91	39.85 ± 3.12
11	4 (0)	10 (−1)	85 (+1)	1.57 ± 0.00	1.42 ± 0.04	0.60 ± 0.01	0.22 ± 0.00	0.19 ± 0.00	11.35 ± 0.06	12.96 ± 2.04	163.09 ± 0.00	31.60 ± 3.69
12	4 (0)	30 (+1)	85 (+1)	1.68 ± 0.01	2.03 ± 0.03	0.90 ± 0.02	0.31 ± 0.00	0.22 ± 0.00	12.65 ± 0.50	10.79 ± 1.99	143.68 ± 0.23	35.86 ± 1.85
13	4 (0)	20 (0)	60 (0)	2.66 ± 0.34	2.70 ± 0.00	1.29 ± 0.00	0.36 ± 0.00	0.28 ± 0.00	17.12 ± 0.00	17.41 ± 0.00	194.12 ± 6.75	40.40 ± 1.93
14	4 (0)	20 (0)	60 (0)	2.09 ± 0.06	2.52 ± 0.00	1.23 ± 0.00	0.32 ± 0.00	0.26 ± 0.00	16.31 ± 0.00	18.19 ± 0.00	192.26 ± 9.80	50.57 ± 2.47
15	4 (0)	20 (0)	60 (0)	2.01 ± 0.02	2.19 ± 0.03	1.06 ± 0.02	0.27 ± 0.00	0.24 ± 0.00	16.52 ± 0.00	18.67 ± 0.00	204.31 ± 2.69	57.36 ± 0.82

t: Extraction time (min); *v*/*w*: Liquid-to-solid ratio (mL/g); EtOH: ethanol concentration (*v*/*v* %); TMAC: Total monomeric anthocyanin content; TAC: Total anthocyanin content (sum of quantified anthocyanins); Peo-3-ch: Peonidin-3-caffeoyl-p-hydroxybenzoyl sophoroside-5-glucoside; Cy-3-ch: Cyanidin-3-caffeoyl-p-hydroxybenzoyl-sophoroside-glucoside; Peo-3-h: Peonidin-3-hydroxybenzoylsophoroside-5-glucoside; TR: Traces; ND: Non detected.

**Table 3:** Regression coefficients and statistical parameters measuring the correlation and significance of the models.

	TMAC	TAC	Peo−3−ch	Cy−3−ch	Peo−3−h	ABTS	DPPH	FRAP	ORAC
βo	2.25 (0.001)	2.47 (0.000)	1.19 (0.000)	0.32 (0.000)	0.26 (0.000)	17.71 (0.000)	16.5 (0.000)	196.90 (0.000)	47.7 (0.000)
β1	0.31 (0.148)	0.48 (0.032)	0.23 (0.028)	0.07 (0.029)	0.04 (0.060)	1.52 (0.034)	1.96 (0.015)	25.25 (0.045)	2.91 (0.227)
β2	0.23 (0.263)	0.31 (0.115)	0.14 (0.12)	0.05 (0.086)	0.03 (0.144)	2.39 (0.006)	1.49 (0.040)	19.08 (0.100)	6.18 (0.033)
β3	0.48 (0.045)	0.58 (0.016)	0.28 (0.014)	0.12 (0.005)	0.02 (0.239)	0.68 (0.248)	0.50 (0.398)	22.82 (0.061)	0.16 (0.942)
β12	−0.04 (0.877)	−0.02 (0.929)	−0.02 (0.895)	−0.01 (0.759)	0.00 (0.959)	1.19 (0.167)	0.12 (0.880)	−1.24 (0.930)	0.40 (0.899)
β13	0.43 (0.149)	0.59 (0.050)	0.28 (0.051)	0.11 (0.027)	0.05 (0.087)	0.55 (0.490)	1.19 (0.179)	17.20(0.256)	2.54 (0.435)
β23	0.01 (0.965)	0.18 (0.468)	0.10 (0.400)	0.03 (0.478)	0.00 (0.952)	−0.77 (0.345)	−1.98 (0.049)	−20.08 (0.195)	−3.00 (0.361)
β11	−0.22 (0.450)	−0.11 (0.658)	−0.04 (0.748)	−0.02 (0.660)	−0.02 (0.462)	−1.22 (0.173)	−1.47 (0.122)	−26.00 (0.121)	−3.77 (0.279)
β22	−0.02 (0.932)	0.02 (0.928)	0.00 (0.984)	0.000 (0.897)	0.01 (0.804)	−1.50 (0.108)	−0.46 (0.585)	−1.69 (0.908)	−2.13 (0.523)
β33	−1.21 (0.006)	−1.46 (0.002)	−0.76 (0.001)	−0.19 (0.003)	−0.100 (0.009)	−5.47 (0.001)	−5.25 (0.001)	−72.46 (0.003)	−12.85 (0.009)
R^2^	0.877	0.933	0.941	0.938	0.87	0.947	0.939	0.909	0.858
CV	13.02	10.73	10.47	12.52	10.23	4.81	5.33	7.86	7.24

TMAC: Total monomeric anthocyanin content; TAC: Total anthocyanin content (sum of quantified anthocyanins); Peo-3-ch: Peonidin-3-caffeoyl-p-hydroxybenzoyl sophoroside-5-glucoside; Cy-3-ch: Cyanidin-3-caffeoyl-p-hydroxybenzoyl-sophoroside-glucoside; Peo-3-h: Peonidin-3-hydroxybenzoylsophoroside-5-glucoside; R^2^: Coefficient of multiple determination; CV: coefficient of variation (%).

**Table 4:** Secondary-polynomial model equations.

(eq 1)	$TMAC=2.25+0.48x_{3}-1.21x_{3}^{2}$
(eq 2)	$TAC=2.47+0.48x_{1}+0.58x_{3}-1.46x_{3}^{2}+0.59x_{1}x_{3}$
(eq 3)	Peo-3-ch $=1.19+0.23x_{1}+0.28x_{3}-0.76x_{3}^{2}$
(eq 4)	Cy-3-ch $=0.32+0.074x_{1}+0.12x_{3}-0.19x_{3}^{2}+0.11x_{1}x_{3}$
(eq 5)	Peo-3-h $=0.260-0.100x_{3}^{2}$
(eq 6)	$ABTS=17.71+1.52x_{1}+2.39x_{2}-5.47x_{3}^{2}$
(eq 7)	$DPPH=16.50+1.96x_{1}+1.49x_{2}-5.25x_{3}^{2}-1.98x_{2}x_{3}$
(eq 8)	$\mathrm{FRAP}=196.90+25.25x_{1}-72.46x_{3}^{2}$

x_1_: Extraction time (min); x_2_: Liquid-to-solid ratio (mL/g) x_3_: ethanol concentration (*v*/*v* %). TMAC: Total monomeric anthocyanin content; TAC: Total anthocyanin content (sum of quantified anthocyanins); Peo-3-ch: Peonidin-3-caffeoyl-p-hydroxybenzoyl sophoroside-5-glucoside; Cy-3-ch: Cyanidin-3-caffeoyl-p-hydroxybenzoyl-sophoroside-glucoside; Peo-3-h: Peonidin-3-hydroxybenzoylsophoroside-5-glucoside.

**Table 5:** Predictive values vs. experimental values.

	Predictive	Experimental	Mean	SD	CV
TMAC (mg/g)	2.50	2.38	2.44	0.1	2.44
TAC (mg/g)	3.15	2.63	2.89	0.26	8.92
Peonidin-3-caffeoyl-p-hydroxybenzoyl sophoroside-5-glucoside (mg/g)	1.51	1.25	1.38	0.13	9.40
Cyanidin-3-caffeoyl-p-hydroxybenzoyl-sophoroside-glucoside (mg/g)	0.42	0.35	0.39	0.04	9.59
Peonidin-3-hydroxybenzoylsophoroside-5-glucoside (mg/g)	0.32	0.28	0.30	0.02	6.92
ABTS (mg TE/g)	20.08	18.78	19.43	0.7	3.36
DPPH (mg AAE/g)	18.13	18.17	18.15	0.0	0.12
FRAP (µmol Fe^2+^/g)	212.29	254.26	233.27	21.0	9.00
ORAC (mg TE/g)	51.29	60.26	55.77	4.5	8.04

SD: Standard deviation; CV: coefficient of variation; TMAC: Total monomeric anthocyanin content; TAC: Total anthocyanin content(Sum of total anthocyanin quantified).

**Table 6:** Anthocyanin profile (µg/g PSPP) comparison between sonotrode-UAE vs. magnetic agitation as optimal extraction conditions: 6 min; 30 mL/g; 60% EtOH from purple sweet potato peel.

	Sonotrode-UAE	Magnetic Agitation
Cyanidin-3-sophoroside-glucoside	28.00 ± 1.46 ^a^	27.69 ± 1.69 ^a^
Peonidin-3-sophoroside-5-glucoside	33.88 ± 1.13 ^a^	31.34 ± 1.91 ^a^
Cyanidin-3p-hydroxybenzoylsophoroside-5-glucoside	133.95 ± 2.34 ^a^	133.95 ± 2.34 ^a^
Cyanidin-3-(6‴-caffeoyl sophoroside)-5-glucoside	2.52 ± 0.16 ^a^	2.63 ± 0.14 ^a^
Peonidin-3-hydroxybenzoylsophoroside-5-glucoside	275.11 ± 9.8 ^a^	251.07 ± 15.73 ^a^
Cyanidin-3-feruloyl sophoroside-5-glucoside	43.09 ± 1.67 ^a^	39.81 ± 3.1 ^b^
Peonidin-3-feruloyl sophoroside-5-glucoside	56.9 ± 4.44 ^a^	49.48 ± 2.13 ^b^
Peonidin-3-caffeoyl sophoroside-5-glucoside	118.15 ± 7.87 ^a^	97.64 ± 5.64 ^b^
Peonidin derivative 5	27.48 ± 2.92 ^a^	23.29 ± 0.57 ^b^
Cyanidin-3-caffeoyl-p-hydroxybenzoyl-sophoroside-glucoside	348.12 ± 9.82 ^a^	312.59 ± 6.8 ^b^
Cyanidin-3-caffeoyl-feruloyl sophoroside-5-glucoside	91.86 ± 1.44 ^a^	80.13 ± 1.58 ^b^
Peonidin-3-caffeoyl-p-hydroxybenzoyl sophoroside-5-glucoside	1253.63 ± 30.46 ^a^	1009.88 ± 21.31 ^b^
Peonidin-3-caffeoyl-feruloyl sophoroside-5-glucoside	202.59 ± 3.41 ^a^	156.7 ± 2.03 ^b^
Peonidin-3-(6′-p′-hydroxybenzoyl-6″-feruloyl sophoroside)-5-glucoside	39.91 ± 1.15 ^a^	31.31 ± 1.26 ^b^
Glycosilated	61.88 ± 2.34 ^a^	59.03 ± 3.57 ^a^
Monoacylated	629.73 ± 16.03 ^a^	568.24 ± 32.49 ^b^
Diacylated	1936.11 ± 44.69 ^a^	1590.61 ± 30.35 ^b^
TAC	2627.72 ± 61.69 ^a^	2217.88 ± 65.06 ^b^
DPPH (mg TE/g)	18.17 ± 0.31 ^a^	10.41 ± 0.4 ^b^
ABTS (mg AAE/g)	18.78 ± 0.48 ^a^	14.92 ± 0.61 ^b^
FRAP (µmol Fe^2+^/g)	254.26 ± 7.93 ^a^	172.98 ± 14.43 ^b^
ORAC (mg TE/g)	60.26 ± 4.53 ^a^	48.31 ± 1.86 ^b^

^a–b^ Mean values in the same row with different letter indicate significant differences (*p* < 0.05) among extraction methods.

## Data Availability

The original contributions presented in this study are included in the article/Supplementary Materials. Further inquiries can be directed to the corresponding author.

## Supplementary Materials

The following supporting information can be downloaded at: https://www.mdpi.com/article/10.3390/foods14152686/s1, Table S1: Effect of sonotrode-UAE on anthocyanin profile under experimental conditions.

## Appendix A

**Table A1:** Anthocyanins identified in purple sweet potato peel.

Peak nº	RT (min)	Formula	Tof MS (m/z)	MS/MS (Fragment Ions (m/z))	Identification
1	13.29	C_33_H_41_O_21_	773.2141	612.1654/450.1107/287.0550	Cy-3-soph-glc
2	17.07	C_34_H_43_O_21_	787.2296	625.1765/463.1239/301.0707	Peo-3-soph-5-glc
2	26.03	C_40_H_45_O_23_	893.2342	731.1807/449.1077/287.0550	Cy-3-*p*-hydroxybenzoylsoph-5-glc
3	27.55	C_42_H_47_O_24_	935.2443	773.1904/449.1086/287.0544	Cy-3-(6‴-caffeoyl soph)-5-glc
4	29.72	C_41_H_47_O_23_	907.2512	745.1976/463.1240/301.0707	Peo-3-hydroxybenzoylsoph-5-glc
5	32.36	C_43_H_49_O_24_	949.2608	787.2059/449.1073/287.0542	Cy-3-feruloyl soph-5-glc
6	35.48	C_42_H_47_O_24_	935.2457	773.1926/449.1074/287.0552	Cy-3-caffeoyl soph-5-glc
7	35.90	C_44_H_51_O_24_	963.2778	801.2241/463.1238/301.0707	Peo-3-feruloyl soph-5-glc
8	38.09	C_43_H_49_O_24_	949.2616	787.2078/463.1233/301.0703	Peo-3-caffeoyl soph-5-glc
9	40.21	C_51_H_53_O_27_	1097.2761	935.2227/449.1055/287.0549	Cy-3-dicaffeoyl soph-5-glc
10	40.31	C_49_H_51_O_26_	1055.2672	893.2138/449.1079/287.0555	Cy-3-caffeoyl-*p*-hydroxybenzoyl-soph-glc
11	44.11	C_52_H_55_O_27_	1111.2934	949.2386/449.1077/287.0547	Cy-3-caffeoyl-feruloyl soph-5-glc
12	45.21	C_52_H_55_O_27_	1111.2928	949.2389/463.1229/301.0698	Peo-3-dicaffeoyl-soph-5-glc
13	45.23	C_50_H_53_O_26_	1069.2793	907.2286/463.1233/301.0698	Peo-3-caffeoyl-*p*-hydroxybenzoyl soph-5-glc
14	46.35	C_49_H_51_O_25_	1039.2695	877.2158/433.1117/271.0597	Pg-3-caffeoyl-*p*-hydroxybenzoyl soph-5-glc
15	49.31	C_53_H_57_O_27_	1125.3080	963.2550/463.1235/301.0698	Peo-3-caffeoyl-feruloyl soph-5-glc
16	52.57	C_51_H_55_O_26_	1083.2977	921.2438/463.123/301.0706	Peo-3-(6′-*p*′-hydroxybenzoyl-6″-feruloyl soph)-5-glc
17	13.29	C_33_H_41_O_21_	773.2141	612.1654/450.1107/287.0550	Cy-3-soph-glc

Peak identification based on Lee et al. [42] and Zhu et al. [45].
